# Using Circadian Rhythm Patterns of Continuous Core Body Temperature to Improve Fertility and Pregnancy Planning

**DOI:** 10.5334/jcr.200

**Published:** 2020-09-24

**Authors:** Wade W. Webster, Benjamin Smarr

**Affiliations:** 1 Prima-Temp Inc., Boulder, CO, US; 2 Department Bioengineering and HDSI, UCSD, San Diego, CA, US

**Keywords:** circadian, menstrual rhythm, core temperature, estrogen, fertility, hypothalamus, progesterone, infradian

## Abstract

**Objective:**

Review relationships among circadian clocks, core body temperature (CBT), and fertility in women.

**Methods:**

Scoping literature review.

**Results:**

Circadian clocks are a ubiquitous adaptation to the most predictable environmental events – the daily cycles of light and dark. Core body temperature (CBT) also follows a circadian rhythm. Additionally, CBT is tightly controlled by a combination of neuronal circuits that begin in the hypothalamus and involve many other portions of the brain as well as a wide range of peripheral mechanisms. In women with normal reproductive function, the diurnal temperature pattern for CBT is strongly influenced by the menstrual cycle of reproductive hormones, primarily estradiol and progesterone, which modulate the activity of hypothalamic neural circuits involved in body temperature control, resulting in an infradian CBT rhythm.

**Conclusions:**

Analysis of CBT via continuous recording reveals patterns in the interactions of circadian and infradian CBT rhythms capable of accurately predicting the fertility window and hormonal patterns suggesting oligo-ovulation and subfertility. New wearable technologies can facilitate employment of hormone-associated changes in CBT for pregnancy planning and offer clinical insight to infertility and menopause.

## Introduction

Continuous core body temperature (CCBT) is an information-rich signal with circadian, infradian, and ultradian rhythms superimposed by dynamic events [1]. Circadian rhythms are self-sustained endogenous rhythms that have approximately 24-hour periods, while infradian rhythms are endogenous rhythms with longer than 24-hour periods; the menstrual cycle is the best known example. In contrast, ultradian rhythms are endogenous cycles with a period shorter than a day. Measurement and analysis of CCBT can provide insight into reproductive health through and beyond menopause. Work carried out more than 20 years ago provided detailed information regarding circadian changes in CCBT that occur across the menstrual cycle in women [2,3]. It was shown that the effects of hormones on circadian patterns and rhythms were so robust that the pattern was easily quantifiable in ambulatory women who were not subjected to controlled lighting, sleep/wake patterns, or activity [2]. This analysis also indicated that assessment of CCBT would be useful for prediction of a “fertility window” for women attempting to become pregnant.

This paper reviews biologic pathways that establish and influence CCBT with a focus on effects of hormones related to the female reproductive cycle. It also reviews use of CCBT to predict fertility and technologies that have been developed to facilitate its monitoring.

## The Biology of Circadian Rhythms

Circadian clocks are found in the vast majority of life forms on Earth and appear to be a ubiquitous adaptation to allow anticipation of predictable environmental changes in sun exposure [4]. Prominent daily rhythms in behavior, physiology, biochemistry, and gene expression are all reflections of organisms’ ability to “keep and tell time” [4] to align with environmental rhythms of day and night. It has been suggested that such clocks may provide advantages with respect to many activities, including avoidance of predation and finding mates and food [5,6].

### Cellular Clocks

Circadian oscillators are present in individual cells and are generated by a set of genes forming a transcriptional autoregulatory feedback loop. In mammals, these “clock genes” include *Clock, Bmal1, Per1, Per2, Cry1*, and *Cry2*. Other candidate genes have also been identified and have been shown to play additional roles in the circadian gene network [7]. Cellular clocks dictate rhythms of approximately 24 hours for many physiological processes including metabolism, division, and death [8,9]. Every circadian clock has the following three characteristics: 1) they oscillate with a period that is close to, but not exactly, 24 hours in duration, so that the clock must be “reset” every day; 2) while chemical reactions run faster as they get hotter, the molecular machinery driving circadian oscillations is buffered against this change so that the clocks run at roughly the same rate across a wide range of environmental temperatures (note this does not preclude using daily temperature changes as a timing cue); and 3) the rhythm will entrain to a rhythmic signal capable of entraining circadian clocks referred to as a zeitgeber [10].

### Neural Circuitry of the Mammalian Biological Clock

Early work on mammalian rhythms indicated that the hypothalamic suprachiasmatic nucleus (SCN) was the dominant circadian pacemaker [11]. This nucleus is composed of approximately 20,000 neurons, each of which is thought to contain a cell autonomous circadian oscillator [7,12]. When dispersed in culture, individual SCN neurons can maintain cell-autonomous circadian cycles of spontaneous firing. However, these autonomous rhythms vary substantially from cell to cell. When SCN circuit connectivity is preserved in slice preparations, neuronal firing is tightly synchronized [12].

The circadian oscillators in the SCN differ from those in other organs in two important ways. First, they receive light input from the eye via retinal ganglion cells that send axons into the retinal–hypothalamic tract. This input is the primary zeitgeber for the circadian pattern of neuronal activity in the SCN [13,14]. The second characteristic of SCN neurons which differentiates them from other cellular oscillators is that their firing pattern, when normal interconnections are intact, is temperature insensitive [15] (though this appears dependent on network-level buffering – see [16]). Other peripheral oscillators are sensitive to the phase-shifting effects of temperature and can be entrained strongly by low amplitude temperature cycles [15,17].

### Influence of the SCN on Peripheral Circadian Rhythms

The light-entrained SCN sends signals to light-insensitive peripheral clocks and synchronizes rhythms across organ systems. Results from multiple studies have shown that both humoral and non-humoral pathways are important for SCN synchronization [18,19]. Though less well studied, there are also feedback loops linking peripheral organs back to the SCN [1,20,21,22,23,24,25,26]). Overall, organization of the circadian system requires autonomic innervation of peripheral tissues, endocrine signaling, temperature sensing, and local signals [7].

### Circadian Rhythms and Core Body Temperature (CBT)

The circadian rhythm of CBT is a well-documented physiological phenomenon [27]. One of the hallmarks of the body’s circadian processes, including cycles in CBT, is that they are not generally disturbed by large temperature changes [9]. This is due to homeostatic control over CBT exerted by a hierarchically organized set of neuronal mechanisms located in the hypothalamus [27]. The anterior hypothalamic and preoptic areas are the primary sources of neural modulation of CBT. These portions of the hypothalamus receive input from both central and peripheral thermoreceptors and from the SCN [27,28]. Two components are essential for their operation: 1) peripheral oscillators in organs other than the brain sensitive to subtle variations in temperature within the physiologic range; and 2) the SCN itself must be resistant to subtle temperature changes or it would be susceptible to feedback that could interfere with entrainment [15]. The insensitivity of the SCN to variations in temperature was demonstrated in studies carried out by Brown et al who also showed that changes in ambient temperature can shift the phase of rhythms in other parts of the brain [17]. The actions of this system result in human diurnal temperature rhythms that typically have a variation of only 1°C (36–37°C) despite large ambient temperature variations [29]. This tight homeostatic regulation is achieved via SCN-driven feedback mechanisms controlling heat production (e.g., via brown adipose tissue and shivering thermogenesis) and loss (e.g., via vasoconstriction and dilation, sweating, and resulting evaporation and cooling) [28].

The above-described mechanisms result in a circadian pattern in which CBT falls late in the activity phase and starts to rise before the onset of the daily activity phase that is highly dependent on both internal zeitgebers, external zeitgebers, and genome-dependent chronotype. Along with the timing of food intake, social interaction, and locomotor activity, internal temperature is one of the most important internal zeitgebers (second-order signals) capable of synchronizing different body clocks [30]. CBT pattern is a reliable signal when compared to melatonin and cortisol production [31]. When measured continuously, the core temperature pattern is an accurate biomarker for circadian phase [32].

### Individual Variation in CBT

Endogenous circadian rhythmicity in humans tends to cycle across a period of 24.2 hours with little day-to-day variation [33]. The timing of the circadian system, however, varies considerably across individuals. Chronotype, or diurnal preference, refers to behavioral manifestations of the endogenous circadian system that govern preferred timing of sleep and waking [34,35]. Variations in normal patterns of circadian rhythms are linked to disease development and chronotypes have received attention across a wide range of illnesses [35]. Chronotypes have a genetic basis and genome-wide association studies have identified multiple genes consistently associated with individual circadian rhythms [35,36].

### Environmental Influence on CBT

Environmental factors also influence individual circadian rhythms. Frequent flying, working in repeatedly changing shifts, and exposure to irregular light-dark conditions are examples of factors that can alter circadian rhythms including that for CBT [37,38].

## Biologic Clocks and Reproduction

**Figure 1:**
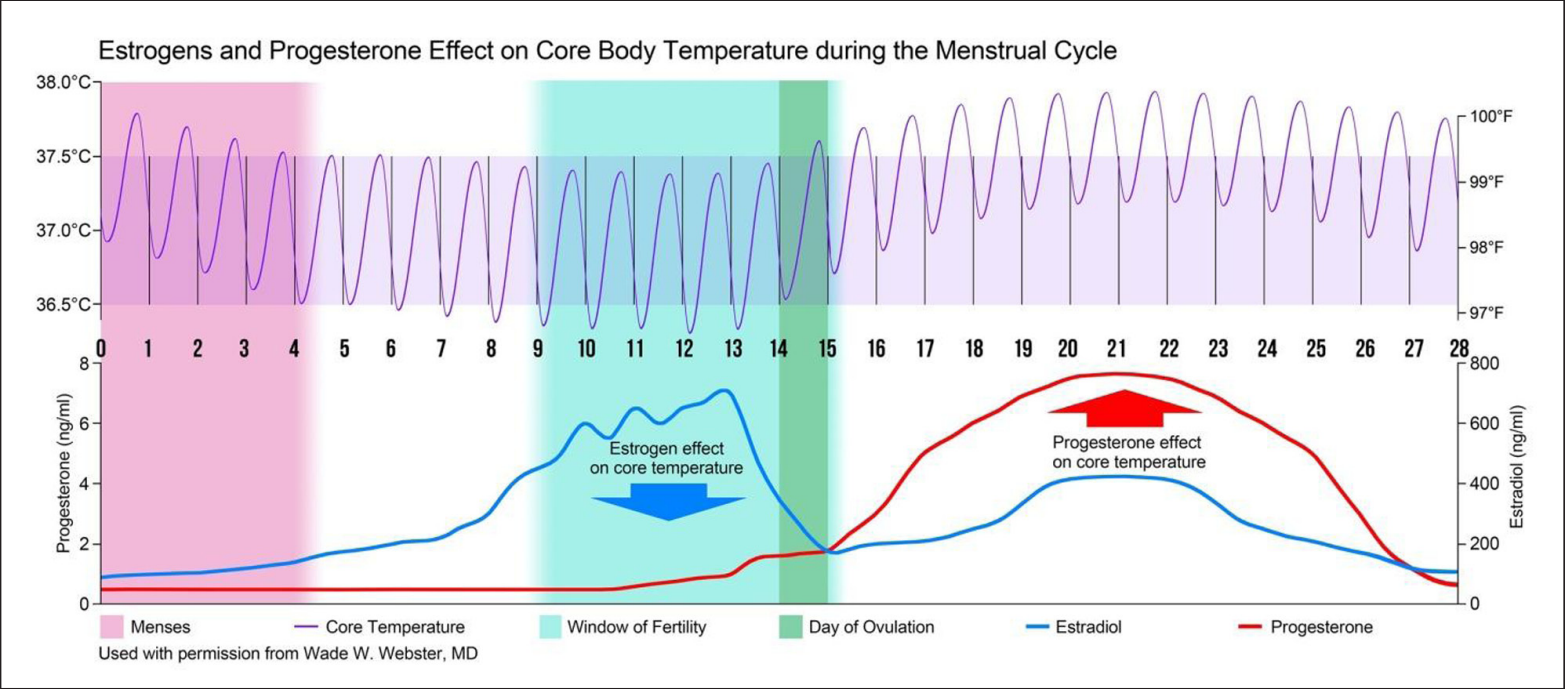
Continuous core body temperature (CCBT) collected during the menstrual cycle reveal an infradian rhythm and pattern. The fine purple line represents a cosinor fit of continuous core body temperature influenced by the combined effect of estrogen and progesterone. Arrows indicate the predominate influence of estrogen in the peri-ovulatory period of the follicular phase and the predominate influence of progesterone in the luteal phase. Analysis of the change in phase, amplitude and mesor of CCBT predict and identify the window of fertility and day of ovulation and mensus.

Reproductive function is a prime example of physiologic coordination and is under strict circadian control [39,40]. In women with normal reproductive function, the diurnal temperature pattern for CBT is strongly influenced by the menstrual cycle of reproductive hormones, primarily estradiol and progesterone. The superimposed effect of reproductive hormone on circadian rhythm that reflects the infradian CBT pattern of the female reproductive cycle illustrated in Figure 1. In the preovulatory portion of the follicular phase there is a downward shift of the temperature mesor associated with an increase in estradiol. This is followed by the upward shift of the temperature mesor as well as a phase shift in temperature related to the rise in progesterone in the luteal phase that may be ratio dependent with respect to its effect on thermoregulation [2,3,41,42].

There is clear evidence of synchronization between the infradian menstrual cycle and the circadian rhythm in reproduction in women. For example, the luteinizing hormone (LH) surge generally occurs immediately prior to the start of the day, while the onset of parturition generally occurs around 2 am to 6 am as a result of the circadian secretion of the pineal hormone – melatonin [41,42,43,44]. Regular, normal length (21–35 days) menstrual cycles are considered a vital sign representing women’s wellness [45,46]. Irregularities in cycle length, such as inadequate luteal phase, are thought to be related to infertility and, potentially, early term miscarriage [47]. Thyroid disease and hyperprolactinemia are causes of infertility that are associated with an inadequate luteal phase [45]. Irregular menstrual cycles, a hallmark of polycystic ovary syndrome, have been associated with higher androgen and lower sex hormone binding globulin levels. This abnormal hormonal environment may also increase the risk of specific histologic subtypes of ovarian cancer [48].

Disruption of circadian rhythms due to night shift work or jet lag has been associated with an increased frequency of irregular, extended menstrual cycles, alterations in serum LH and follicle-stimulating hormone (FSH) levels, and overall reduced fecundity [49,50,51]. Polymorphisms in genes that control circadian rhythm have also been associated with the rate of pregnancy loss and risk for miscarriage [52].

## Continuous Assessment of CBT (CCBT) and Utility of Menstrual Rhythm Temperature Patterns

The sinusoidal oscillations in CCBT can be mathematically described on the basis of amplitude, mesor, and period. Using CBT, Cagnacci et al, characterized the follicular phase by a 0.3 °C lower mesor, a 40% increase in the amplitude and a 90-minute advance in the daily nadir compared to the luteal phase [53]. Signal processing analysis of CCBT data has been demonstrated to be effective for detecting and predicting physiologic events of fertility, including ovulation, pregnancy, parturition, and spontaneous abortions [54,55,56,57].

As noted in the **Introduction**, the predictive value of the information contained in the infradian pattern of body temperature in women was clearly demonstrated by results from studies reported by Coyne et al 20 years ago [2]. These investigators carried out a detailed investigation of circadian changes in body temperature occurring across the menstrual cycle in women. Sensors were ingested to accurately monitor CBT once per minute. After calculation of the mesors for circadian temperatures during different parts of the menstrual cycle, it was observed that the circadian mesor for core body temperature is highest in the luteal phase and lowest in the preovulatory phase. The amplitude of circadian temperature was significantly reduced in the luteal phase compared to all times in the menstrual cycle. The effects of hormones on the circadian pattern and rhythm for body temperatures were readily apparent and easily quantifiable in ambulatory women who were not subjected to controlled lighting, sleep/wake patterns, or activity. Coyne et al noted that their observations supported documenting the preovulatory rise in estrogens via a decline in CBT, which would in turn provide an opportunity to investigate phenomena that might be occurring during this critical time in the cycle [2].

### Detection of Pregnancy

Pregnancy was shown to cause a change in the pattern of body temperature rhythms in 1948 [58], and has since then been remarkably unvisited. Smarr used CCBT frequency analysis for pregnancy detection in mice within 14 hours of pairing, providing the earliest, non-disruptive detection of successful impregnation [59]. This approach was also able to detect apparent pregnancies that did not come to term, which would otherwise not be identified by standard handling or observation, providing a potential source of dams for the study of implantation failure, pseudopregnancy, and miscarriage. Such pregnancies could be separated from those that came to term by frequency analysis of CCBT in the first 12 hours after the day pairing with high accuracy. These findings support the conclusion that the continuous high temporal resolution of CCBT can provide a uniquely rapid, accurate, and non-disruptive means of detecting pregnancies and pregnancy outcomes in experimental animals [59].

### Prediction of Fertility

The biphasic basal body temperature (BBT) rhythm during the menstrual cycle reaches its lowest point in a given cycle around the woman’s fertile window, just prior to ovulation, which is correlated with a pre-ovulatory peak in estrogens level illustrated in Figure 1 [60]. Changes in the level of estrogens, as well as progesterone, influence body temperature via a direct action on thermosensitive neurons of the preoptic anterior hypothalamus [61,62]. Estrogens lead to inhibition of mechanisms that act to retain heat and stimulation of those that promote heat loss. Thus, elevations in estrogens result in a reduction in body temperature [63,64]. Progesterone has opposing effect and the rise in this hormone post-ovulation increases body temperature [64,65]. The thermogenic effect of progesterone to raise core temperature has been utilized to document ovulation and luteal phase length [66,67].

Silent anovulation is a significant underreported problem. It is defined as sporadic anovulation in women with regular menses and has a prevalence up to 37% in community-based cohorts [67]. This is especially relevant for clinical care as the current methods used for monitoring menstrual cycles are widely believed to be inconvenient and cumbersome. The prevalence of silent anovulation in more than one-third of clinically normal menstrual cycles represents a major knowledge gap for improving understanding of women’s reproductive physiology [67]. Monophasic patterns that lack the thermogenic effect of progesterone suggest anovulation as opposed to the biphasic pattern that confirms ovulation [66]. It is increasingly evident that silent anovulation within clinically normal menstrual cycles is relevant for women’s health as well as for fertility. Prior et al have shown that subclinical ovulatory disturbances are associated with annual increased spinal bone loss. [68]. Ovulatory disturbances are also related to women’s risks for later-life heart disease and likely also to breast and endometrial cancer risks [69,70,71].

## New Approaches to CCBT May Facilitate Fertility Prediction

The ability to detect fertility has become increasingly important due to changing approaches to family planning. As women delay childbearing, couples may have an increased sense of urgency when starting a family. In addition, increased exposure to disruptive environmental elements, such as high calorie diets, increasing obesity and lack of exercise, prolonged periods of artificial lighting, shift work, trans-meridian travel, jet lag, and disordered sleep, all have the potential to impair fertility [13].

Basal body temperature (defined as once-a-day temperature measurement at same time of day) charting has been used extensively as a simple aid for predicting ovulation [72]. Standard practice is to measure BBT upon waking, but as this is a single time point and not the nadir of CBT, BBT data do not precisely capture changes in circadian temperature rhythms seen across the menstrual cycle. However, many currently available products and tracking tools require a considerable amount of user interaction to record frequent temperature readings, urine test results, and other observations. This can lead to frequent errors, user stress. and frustration [72,73].

New wearable technologies are capable of recording core body temperature and wirelessly pair to smartphones allowing sophisticated analysis of continuous recording of body temperature [73,74]. Other approaches have included shell or peripheral temperature measurement [72]. While there are no head-to-head comparisons of devices employing these different approaches, it has been suggested that assessment of skin temperature may not be an accurate predictor of ovulation [75]. For example, results obtained with a wrist temperature sensor indicated sustained 3-day temperature shift in only 82% of cycles and the lowest cycle temperature occurred in the fertile window 41% of the time. In addition, most temporal shifts (87%) occurred on ovulation day or later [76].

Over the last several decades, BBT has been used to identify the biphasic temperature change that verifies ovulation and that information has been applied to the next cycle assuming that each cycle is as regular as the first. Obviously, this does not account for individual variability in a woman’s menstrual cycle each month. Identifying the more subtle periovulatory infradian temperature nadir is fraught with the inaccuracy of a single once-a-day peripheral temperature measurement. More importantly, research does not support the use of the BBT nadir for predicting ovulation [77]. Additionally, BBT is not able to make use of the information found in higher-frequency measurements of body temperature now being discovered.

CCBT can provide accurate and precise prediction of ovulation in addition to identifying the highest probability within the window of fertility [74]. Additionally, true CCBT is collected night and day to catch changes that may occur at any time in a 24-hour period [78], including true mesor, and higher frequency components, as in ultradian rhythms. Temperatures obtained from an intravaginal device identical in form to intravaginal rings currently sold on the market provides a safe, continuous and accurate CBT as well as real-time access to sophisticated algorithms that can process an alert or notification to a smartphone. The convenience of no longer tracking a potentially unreliable peripheral temperature at the same time of early morning hours is evident.

## Conclusions

Access to continuous CBT provides information not usually available to clinicians, but it is capable of predicting changes across menstrual cycles and classifying etiologies of infertility. CBT follows a distinct circadian rhythm and is also influenced by reproductive hormones which interact with hypothalamic neural circuits. Signal processing analyses applied to a precise, accurate and continuous CBT combined with new wearable technologies can accurately classify CBT changes for use in fertility awareness, infertility, silent anovulation, and a number of other health-related applications. The more immediate benefit of CBT is to help better define the window of fertility for the purposes of avoiding or promoting conception.
